# A Non-Traditional Model of the Metabolic Syndrome: The Adaptive Significance of Insulin Resistance in Fasting-Adapted Seals

**DOI:** 10.3389/fendo.2013.00164

**Published:** 2013-11-01

**Authors:** Dorian S. Houser, Cory D. Champagne, Daniel E. Crocker

**Affiliations:** ^1^Department of Conservation and Biological Research, National Marine Mammal Foundation, San Diego, CA, USA; ^2^Department of Biology, Sonoma State University, Rohnert Park, CA, USA

**Keywords:** metabolic syndrome, elephant seal, insulin resistance, fasting, hyperglycemia

## Abstract

Insulin resistance in modern society is perceived as a pathological consequence of excess energy consumption and reduced physical activity. Its presence in relation to the development of cardiovascular risk factors has been termed the metabolic syndrome, which produces increased mortality and morbidity and which is rapidly increasing in human populations. Ironically, insulin resistance likely evolved to assist animals during food shortages by increasing the availability of endogenous lipid for catabolism while protecting protein from use in gluconeogenesis and eventual oxidation. Some species that incorporate fasting as a predictable component of their life history demonstrate physiological traits similar to the metabolic syndrome during prolonged fasts. One such species is the northern elephant seal (*Mirounga angustirostris*), which fasts from food and water for periods of up to 4 months. During this time, ∼90% of the seals metabolic demands are met through fat oxidation and circulating non-esterified fatty acids are high (0.7–3.2 mM). All life history stages of elephant seal studied to date demonstrate insulin resistance and fasting hyperglycemia as well as variations in hormones and adipocytokines that reflect the metabolic syndrome to some degree. Elephant seals demonstrate some intriguing adaptations with the potential for medical advancement; for example, ketosis is negligible despite significant and prolonged fatty acid oxidation and investigation of this feature might provide insight into the treatment of diabetic ketoacidosis. The parallels to the metabolic syndrome are likely reflected to varying degrees in other marine mammals, most of which evolved on diets high in lipid and protein content but essentially devoid of carbohydrate. Utilization of these natural models of insulin resistance may further our understanding of the pathophysiology of the metabolic syndrome in humans and better assist the development of preventative measures and therapies.

## Introduction

Dyslipidemia, altered glucose metabolism, and hypertension are all hallmark characteristics of the metabolic syndrome that are often, but not always, associated with obesity. It has been argued that the logical predecessor that binds these cardiovascular risk factors together is insulin resistance (1). Insulin resistance, however, is not uniform across the tissues of an organism and there is obvious differentiation in insulin resistance between hepatic, adipose, and muscle tissues. There is also considerable debate over the causative nature and interaction of factors contributing to insulin resistance with lipid partitioning, adipocytokines (particularly adiponectin), and the contribution of reactive oxygen species at the forefront of the discussion. Treatment and prevention of the metabolic syndrome is of increasing concern due to the rapid increase in its occurrence, which exceeds 30% of the adult U.S. population and which shows a continuing upward trajectory (2, 3).

Insulin resistance is today almost ubiquitously viewed as a pathological phenomenon. Ironically, from an evolutionary perspective, insulin resistance likely served as an adaptive response to increased demands for endogenous nutrient stores as might occur during injury or starvation (4, 5). Under such situations, mobilization of fat stores and reductions in the demand for glucose by non-glucose dependent tissues would be facilitated by reductions in insulin signaling. In the modern world, chronic energy surplus combined with reduced physical activity results in excess fat deposition, lipid overflow into non-adipose tissues, and inflammation of adipose tissue, all of which contribute to insulin resistance and increased morbidity (4).

Traditional animal models for the study of the metabolic syndrome and diabetes typically include rodents (e.g., the Zucker diabetic fatty rat or Goto-Kakizaki rat), felines, swine, and primates (6, 7). Model species may naturally exhibit diabetes or the metabolic syndrome or may have diabetic-like states induced via dietary, genetic, or pharmacological manipulations (8). Traditional animal models are typically selected because of the similarity between humans and the animal model with regard to specific pathological traits of interest. However, there is no single traditional animal model which exactly mimics diabetes or the metabolic syndrome in humans and little work has been performed in non-traditional or exotic animal models.

Insulin declines with the progression of fasting in humans, facilitating lipolysis while insulin resistance increases (9, 10). This adaptive feature under the constraint of nutrient deprivation is hardly constrained to humans and appears to be a universal response to food deprivation, at least among mammals. Some mammals incorporate predictable periods of fasting as a component of their life histories and the development of insulin resistance within the progression of a fast is likely critical to their ability to regulate endogenous nutrient use across the fast. The northern elephant seal (*Mirounga angustirostris*) is one of a handful of mammalian species that couples complete fasting from food and water with energetically costly activities such as lactation, intrasexual competition, molting, and post-natal ontogeny. These prolonged fasts occur twice each year in association with the periods that the seals are on land; elephant seals are the most aquatic of the seals and will spend 8–10 months of the year at sea while on foraging migrations. Upon arrival to land, or upon weaning (for pups), up to 50% of the elephant seal’s mass is due to adipose stores, specifically blubber, a non-visceral fat depot. The time fasting on land ranges from ∼25 days (lactating females) to 4 months (sexually competitive males) with intermediate fasting durations experienced by pups, juveniles, and molting seals of all age classes.

Elephant seals demonstrate a moderate fasting hyperglycemia, a muted insulin release in response to a glucose challenge, fasting hyperlipidemia, and peripheral insulin resistance (details discussed below). Although the fasting levels of circulating glucose may not always exceed the clinical threshold typical for diagnosing diabetes, the glucose levels and characteristics of the glucoregulatory system are similar to those of the metabolic syndrome. However, unlike humans in whom the condition is pathological, the shared characteristics of the metabolic syndrome in the northern elephant seal enable it to manage the fate of endogenous nutrients and reproduce and develop under conditions in which it is isolated from food sources for weeks to months. Here we review glucose metabolism and insulin resistance in the northern elephant seal insofar as it parallels and differs from the physiological characteristics of the metabolic syndrome. It is necessary, however, at the outset to distinguish between the different age and reproductive categories of the elephant seal that have been studied as the age and reproductive status strongly influences glucose metabolism within this species. Below, a brief description of the different classes of seal is given. This is then followed by a discussion of what is known about glucose metabolism and insulin resistance in this species, characteristics that make the elephant seal an atypical but potentially useful model of the metabolic syndrome.

### Life history stages

Adult female elephant seals arrive on rookeries in the early winter to give birth and nurse their young. This period is also coupled with breeding and adult males overlap with adult females on land only during this period. Females give birth to pups within several days of arriving on land and nurse them for ∼25 days. Lactating females produce copious amounts of milk; nursing pups may consume in excess of 4 kg of milk per day. The milk produced by elephant seals is one of the most energy rich milks observed in nature. It exceeds 50% lipid content by mass by the middle of lactation and becomes more lipid rich with the progression of lactation (11–13). Protein content remains stable across lactation at ∼10% of the milk composition by mass. The milk contains negligible carbohydrate. Endogenous lipid reserves are mobilized at a high rate to support lactogenesis and maternal maintenance metabolism (14). Adult females may lose up to 57% of their fat mass and 25% of their body protein over the lactation period (13).

Elephant seal pups are abruptly weaned when their mothers return to sea to feed. During the three-and-a-half week lactation period, pups will increase from a birth mass of ∼35 kg to in excess of 120 kg. Body composition at weaning is typically 40–50% lipid, almost all of which is contained within the blubber layer (15). Upon weaning, pups undertake a post-weaning fast that can extend beyond 8 weeks in duration. Pups spend increasing amounts of time in surf zone waters with the progression of the fast in order to learn the skills necessary for swimming and diving at sea. At the same time, endogenous nutrients are mobilized for the *de novo* synthesis of blood and respiratory pigments necessary for extended breath-holds while diving (16).

Adult male elephant seals arrive on land in late autumn to early winter for the breeding season. Combative intrasexual competition between adult males is used to establish a dominance hierarchy that ultimately contributes to breeding access to females. Males may fast from food and water for up to 4 months during the breeding season and may expend a considerable amount of energy in breeding activities. Males may lose nearly a quarter of their body mass prior to returning to sea to feed (17, 18).

Elephant seals return to land either in the spring (females) or summer (males) to molt, following the post-breeding period return to sea to feed. During this “catastrophic” molt the entire pelage is shed and replaced. Although it has not been directly quantified, the production of a new pelage must affect protein mobilization and use and likely places different constraints on glucose metabolism than does lactation or post-weaning development.

## Glucose Production

The final report of the National Cholesterol Education Program (NCEP) presented clinical criteria for defining the metabolic syndrome (19). The NCEP proposed that three of five criteria be met for the clinical diagnosis of the metabolic syndrome. These included thresholds for abdominal obesity, triglyceride levels, cholesterol levels (particularly low high-density lipoprotein, HDL), hypertension, and impaired fasting glucose (≥100 mg/dL, or 6.1 mM). A survey of how impaired fasting glucose alone compared to individuals with metabolic syndrome showed that nearly half of all individuals with impaired fasting glucose also met the criteria for the metabolic syndrome (20). Although there is insufficient information to determine how all of these factors compare to the natural state of the elephant seal, fasting glucose in the elephant seal commonly exceeds the human threshold set by the NCEP. Circulating glucose in fasting elephant seals ranges from 5.5 to 9.8 mM and varies with time fasting and the age class or reproductive state of the seal (Table 1). The highest glucose levels, which typically exceed 8 mM, are observed in elephant seals at the beginning of their post-weaning fast. In this age class, there is a modest decrease in plasma glucose with the progression of the fast. Within lactating females, glucose levels remain relatively constant across the duration of the fast, although the total length of the fast is substantially shorter than that of the weanling seals.

**Table 1:** **Summary information of metabolites and glucoregulatory hormones studied in the different life history stages of the northern elephant seal**.

Class	Glucose (mM)	Lactate (mM)	BUN (mg/dL)	NEFA (mM)	Glycerol (μM)	BHB (μM)	AcAc (μM)	Insulin (pg/mL)	Insulin (pM)	Glucagon (pg/mL)	Glucagon (pM)	I:G	Cortisol (ng/mL)	Cortisol (nM)	EGP (mmol/min)	Reference
**ADULT FEMALE**																
Early lactation	7.0 (0.7)	2.4 (0.3)			189 (49)	264 (24)		101.8 (16.9)	17.5 (2.9)	43.1 (11.4)	12.4 (3.3)	1.5 (0.5)	53.5 (19.6)	147.7 (54.1)	3.9 (0.7)	Champagne et al. (23)
Early lactation	7.4 (0.5)	2.5 (0.8)		1.0 (0.2)	250 (60)	700 (230)		98.2 (20.3)	16.9 (3.5)	57.1 (18.5)	16.4 (5.3)	1.0 (–)	52.2 (24.8)	144.2 (68.4)		Houser et al. (14)
Early lactation	7.0 (0.2)							83.3 (8.8)	14.3 (1.5)	29.8 (2.1)	8.4 (0.6)	1.7 (0.2)				Fowler et al. (24)
Early lactation	7.6 (0.5)	2.4 (0.8)						67.4 (15.1)	11.6 (2.6)	37.3 (13.2)	10.7 (3.8)	1.2 (0.4)	56.9 (12.0)	157 (33)		Champagne et al. (76)
Late lactation	8.2 (0.7)	2.5 (0.4)			462 (171)	345 (40)		68.8 (11.6)	11.8 (2.0)	38.8 (11.4)	11.1 (3.3)	1.2 (0.4)	103.5 (29.9)	285.7 (82.5)	3.3 (0.8)	Champagne et al. (23)
Late lactation	7.2 (1.0)	2.7 (0.7)		3.2 (0.8)	520 (220)	760 (210)		76.7 (18.0)	13.2 (3.1)	54.3 (13.2)	15.6 (3.8)	0.9 (–)	89.4 (30.1)	246.8 (83.2)		Houser et al. (14)
Late lactation	6.8 (0.5)							43.0 (10.1)	7.4 (1.7)	36.6 (5.5)	10.5 (1.6)	0.7 (0.1)				Fowler et al. (24)
Late lactation	7.7 (0.6)	2.4 (0.8)						58.7 (23.8)	10.1 (4.1)	41.1 (10.8)	11.8 (3.1)	0.9 (0.3)	71.4 (17.8)	197 (49)		Champagne et al. (76)
Post-molt	7.1 (0.6)	3.8 (0.8)			204 (82)	288 (52)		87.0 (18.1)	15.0 (3.1)	46.5 (13.4)	13.4 (3.8)	1.2 (0.3)	80.8 (20.7)	223.0 (57.1)		Champagne et al. (23)
Post-molt	6.2 (0.2)	3.7 (1.0)		1.4 (0.3)	340 (80)	630 (80)		86.0 (15.7)	14.8 (2.7)	46.7 (8.0)	13.4 (2.3)	1.1 (–)	107.2 (66.9)	295.8 (184.6)		Houser et al. (14)
Post-molt	7.0 (0.3)							84.2 (5.5)	14.5 (0.9)							Fowler et al. (24)
**ADULT MALE**																
Early fasting	6.0 (0.3)		10.2 (0.3)	0.8 (0.1)		234 (11)		73.9 (3.5)	12.7 (0.6)	35.9 (3.5)	10.3 (1.0)	1.3 (0.1)	83 (7)	229.1 (19.3)		Crocker et al. (17, 33)
Late fasting	5.5 (0.4)		10.5 (0.3)	0.8 (0.6)		365 (12)		68.3 (2.0)	11.8 (0.3)	49.9 (2.9)	14.3 (0.8)	0.8 (0.1)	77 (9)	212.5 (24.8)		Crocker et al. (17, 33)
**WEANLING**																
Early fasting			28.9 (11.5)										49 (17)	135.2 (46.9)		Houser et al. (25)
Early fasting	8.6 (0.9)							67.6 (10.5)	11.6 (1.8)	51.6 (14.0)	14.8 (4.0)	0.8 (0.2)	53.3 (26.1)	147.1 (72.0)	1.6 (0.1)	Champagne et al. (22)
Early fasting	8.9 (1.1)	3.1 (0.7)				423 (87)										Tavoni et al. (29)
Early fasting	8.6 (0.3)	2.5 (0.4)				477 (100)	129 (7)	77.8 (21.5)	13.4 (3.7)	28.9 (4.9)	8.3 (1.4)	1.6 (–)	64.31 (10.4)	177.5 (28.7)	1.4 (0.3)	Houser et al. (15)
Early fasting	7.8 (0.6)	3.7 (0.9)						62.1 (43.6)	10.7 (7.5)	69.3 (15.0)	19.9 (4.3)	0.5 (0.4)	63.4 (23.9)	175 (66)		Champagne et al. (76)
Early fasting	9.7 (0.8)	3.0 (0.8)				56.6 (10.4)		61.6 (13.4)	10.6 (2.3)	44.6 (9.1)	12.8 (2.6)	0.9 (0.3)		564 (238)	1.8 (0.3)	Champagne et al. (21)^a^
Early fasting	9.6 (0.7)			0.9 (0.1)	180 (10)			87.1 (10.5)	15.0 (1.8)				95.7 (12.7)	264 (35)		Viscarra et al. (34 –36)^a^
Early fasting	9.8 (0.5)			0.7 (0.0)				94.1 (13.9)	16.2 (2.4)				44.9 (2.2)	124 (6)		Viscarra et al. (36)^a^
Early fasting																
Late fasting			19.5 (15.6)										98 (63)	270.5 (173.9)		Houser et al. (25)
Late fasting	8.0 (0.5)							57.1 (13.6)	9.8 (2.3)	69.4 (17.0)	19.9 (4.9)	0.5 (0.2)	61.3 (22.7)	169.2 (62.7)	1.1 (0.0)	Champagne et al. (22)
Late fasting	8.5 (1.4)	1.8 (0.6)				997 (169)	170 (16)	53.4 (19.7)	9.2 (3.4)	24.4 (4.5)	7.0 (1.3)	1.3 (–)	65.5 (12.0)	180.7 (33.2)	1.0 (0.2)	Houser et al. (15)
Late fasting	7.5 (0.3)	3.0 (0.8)						45.9 (16.3)	7.9 (2.8)	68.3 (12.5)	19.5 (3.6)	0.5 (0.2)	78.6 (27.2)	217 (75)		Champagne et al. (76)
Late fasting	10.3 (0.8)	2.2 (0.8)				128.6 (19.3)		35.4 (15.1)	6.1 (2.6)	58.5 (19.2)	16.8 (5.5)	0.4 (0.1)		788 (419)	1.2 (0.2)	Champagne et al. (36)^a^
Late fasting	7.1 (0.5)			1.6 (0.2)	240 (10)			41.8 (3.5)	7.2 (0.6)				172.8 (17.8)	477 (49)		Viscarra et al. (34 –36)^a^
Late fasting	8.0 (0.3)			0.9 (0.1)				34.8 (3.5)	6.0 (0.6)				93.1 (10.9)	257 (30)		Viscarra et al. (36)^a^

*BUN, blood urea nitrogen; NEFA, non-esterified fatty acids; BHB, beta-hydroxybutyrate; AcAc, acetoacetate; I:G, insulin to glucagon ratio; EGP, endogenous glucose production*.

*^a^ Certain values are suspect due to the handling artifacts inherent to the study*.

**Figure 1:**
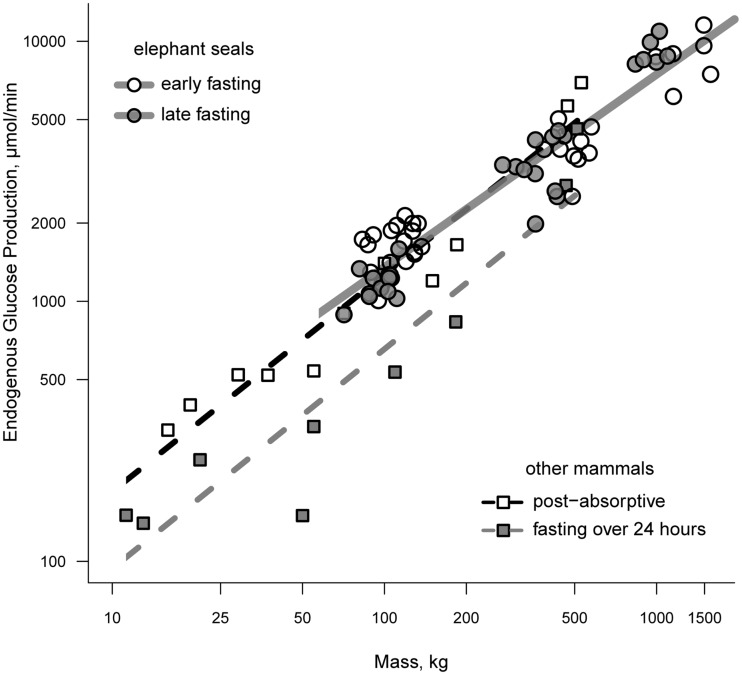
**A comparison of endogenous glucose production between fasting seals and other terrestrial mammals**. Terrestrial mammals are separated as those that are post-absorptive (open squares) and those that have been fasting for 24 or more hours (filled squares). Elephant seals are separated according to whether measurements were made during the early or late fasting period (see text for details on fasting duration as a function of life history stage).

Endogenous glucose production (EGP) has been measured in all classes of elephant seal. Rates of EGP in weanlings range from 1.0 to 1.8 mmol/min between the early and late fasting periods and show of trend of declining EGP with time fasting (15, 21, 22). Total EGP averages between 3.3 and 3.9 mmol/min and trends downward with the progression of lactation in females, although this trend disappears once corrected for body mass (23). Adult females that have recently molted have rates of EGP that are intermediate of early and late lactation values (23). A decline in EGP with time fasting is consistent with the fasting-associated down-regulation of glucose metabolism; however, the rate of glucose production in elephant seals fasting for 1–3 months is comparable to that of post-absorptive animals when controlling for mass (24). Figure 1 demonstrates the relationship between mass and EGP across a broad range of animal masses under post-absorptive and fasting conditions.

Maintaining relatively high levels of EGP across the fast is likely controlled in part by fasting-dependent changes in regulatory hormones and adipocytokines, factors which are consistent with observations of the metabolic syndrome. These factors not only affect specific signaling pathways, but the mobilization of nutrients for glucose synthesis. Recent investigations have shown that EGP in fasting weanlings is not derived from glycogen stores (15, 21). This finding is consistent with an expectation of glycogen depletion over extended fasts. Protein is strictly conserved across the post-weaning fast and the loss of amino acids to gluconeogenesis is minimized (15, 25–27). However, the ability to conserve protein stores is progressively lost across lactation in adult females due to the combined demands of lactation and maintenance metabolism (28). The contribution of glycerol to EGP is sufficient to replace the glucose that is lost to oxidation (see below), but in both adult females and weanlings, glycerol gluconeogenesis contributes <5–10% to EGP (14, 21).

Evidence gathered to date, including the nuclear magnetic resonance (NMR) measurement of pathway flux rates associated with the tricarboxylic acid (TCA) cycle, point to phosphoenolpyruvate (PEP) as the dominant gluconeogenic precursor within fasting elephant seals (21). Indeed, within weanling seals, PEP accounts for ∼95% of EGP and the contribution of PEP to gluconeogenesis declines in direct relation to fasting-related reductions in the rate of EGP. The origin of PEP for gluconeogenesis is likely the lactate-pyruvate pool (29), and it has been hypothesized that high rates of phosphoenol pyruvate carboxykinase (PEPCK) and pyruvate cycle activity exist to facilitate EGP by replacing TCA cycle intermediates without loss of protein (amino acids) to anaplerotic reactions (21).

It has long been known that the metabolic rate of the fasting elephant seal is predominantly met by the oxidation of fat while protein contributes little to meeting the metabolic demand (25–27, 30). Minimal contributions of glucose oxidation to the metabolic rate have historically been determined by subtraction of energy derived from protein and lipid oxidation from the whole animal metabolic rate. However, recent measurements of the respiratory quotient in weanling seals has shown that glucose oxidation contributes <10% to the metabolic rate across the fast (15). Between 16 and 24% of EGP is oxidized and the amount oxidized is tightly coupled to the rate of EGP, which declines with time fasting.

Available pathways for the non-oxidative disposal of glucose consist of glycogen production, lipogenesis, and the production of lactate, pyruvate, and alanine. Although nearly all glucose enters glycolysis, there is no evidence for lipogenesis or the creation of glycogen via the direct pathway through glucose 1-phosphate (15). This suggests that glucose is converted to one of the potential gluconeogenic precursors and subsequently recycled via a 3-carbon intermediate (e.g., lactate), consistent with the findings of Champagne et al. (24) and Tavoni et al. (29).

## Regulation of Glucose Metabolism

### Insulin and insulin resistance

By most accounts the definition of the metabolic syndrome centers around insulin resistance, a factor which will be demonstrated also exists in the fasting elephant seal. However, a critical distinction between the human pathological condition and the adaptive condition of the elephant seal is the occurrence of hyperinsulinemia. In man, the metabolic syndrome and the progression to type 2 diabetes is defined in part by the presence of hyperinsulinemia – first in post-prandial measures, and later during fasting (31). This hyperinsulinemia is, however, never observed in elephant seals. Insulin levels are consistently low across all classes of elephant seal (Table 1). Maximum levels of circulating insulin are <20 pM and fall to ≤13.2 pM during the late portion of the lactating fast and ≤9.8 pM late in the post-weaning fast. This feature is likely adaptive to the extent that it facilitates lipolysis and allows the elephant seal to maximize use of its copious adipose stores for meeting metabolic demands. Numerous mechanisms of how insulin inhibits lipolysis, and how the lack of insulin secretion facilitates lipolysis, have been investigated (32). The reduction in insulin responsiveness to glucose, as well as an increase in insulin resistance, may very well be a conserved feature of the fasting response (4, 5, 9), albeit taken to the extreme in the northern elephant seal.

Reductions in circulating insulin parallel the sensitivity of pancreatic β-cells, at least as measured in lactating females. Early during lactation, a 150-g intravenous injection of glucose resulted in a ∼129% increase in circulating insulin 10 min post-injection (37). No insulin response to the same glucose tolerance test (GTT) was observed during late lactation, whereas post-molt females demonstrated an intermediate response, increasing insulin by ∼62%. Similar types of GTTs have been attempted in pups just prior to weaning and those fasted for 8–11 weeks. The authors reported no insulin response to a 25-g glucose challenge in either nutritional state, i.e., fasting vs. feeding (34, 38). Collectively, these findings suggest pancreatic β-cells have a reduced responsiveness to circulating glucose, either inherently (e.g., genetic) or through variability in epigenetic expression of metabolic control points.

A relationship between the proportion of body mass due to adipose and the degree of insulin response following a GTT has been offered as evidence that depletion of adipose stores relates to functional suppression of the insulin response (37). This explanation is not consistent with the findings of Kirby and Ortiz (38) as pups tested in this study were in both feeding (adipose gaining) and fasting (adipose depleting) states. A more parsimonious explanation may be that a prolonged state of hyperlipidemia, specifically high levels of certain circulating non-esterified fatty acids (NEFAs), contributes to a decline in the ability of pancreatic β-cells to respond to an increase in the glucose load as has been observed both *in vivo* and *in vitro* (39, 40). In female elephant seals, the increase in NEFA from 1.0 to 3.2 mM across the 25-day lactation fast constitutes a progressive and continuous exposure to elevated levels of NEFA that may explain the muted response in glucose stimulated insulin secretion (GSIS). The lack of an insulin response in weanlings measured prior to weaning and after 8–11 weeks of fasting can be explained by a similar mechanism. While nursing, pups are consuming large quantities of lipid and may consume in excess of 4 L of milk per day by the end of lactation, >50% of which is lipid (12, 13, 41). Thus, there is no shortage of NEFA availability while nursing. Conversely, pups continue to be exposed to NEFA levels between 0.7 and 1.6 mM throughout the post-weaning fast. From birth to the end of the fast, pups therefore experience a persistent exposure to high levels of NEFA.

Several lines of evidence also suggest peripheral insulin resistance in both adult female seals and pups. Following a GTT, adult females showed no relationship between a calculated glucose tolerance index and the amount of insulin secreted per minute, although the time fasting and life history stage (lactating vs. molting) had a significant influence over GSIS (37). The insulin sensitivity index, calculated as the ratio of the area under the curve for glucose to that for insulin, also suggested peripheral insulin resistance with a slight increase in resistance with time fasting in the lactating females. Insulin tolerance tests (ITTs) conducted in fasting weanlings and nursing pups are also indicative of peripheral insulin resistance and an increase in peripheral resistance with time fasting (34, 38). Interestingly, with the onset of the post-weaning fast and the cessation of consuming high-fat content milk, it appears that GSIS in the pups may temporarily return to a state more similar to that of non-insulin resistant mammals (34).

Reductions in the phosphorylated forms of insulin receptor (IR), insulin receptor substrate-1 (IRS-1), and expression of the Akt2 gene within adipose tissue and with time fasting are also suggestive of mounting insulin resistance in elephant seal weanlings (35). This apparent reduction in insulin signaling is accompanied by an increase in the expression of the plasma membrane glucose transporter 4 (Glut-4) within the same adipose tissue (35). Since Glut-4 is responsible for the cellular uptake of glucose, the increased glucose uptake by the diminishing adipose depot may partially explain the modest reductions in EGP and circulating glucose concentrations observed between the early and late periods of the post-weaning fast. An increase in the phosphorylation of AMPK within adipose tissue, which is observed late in the fast (34), has been hypothesized as an insulin-independent mechanism by which Glut-4 translocation might facilitate modest reductions in circulating glucose levels. Indeed, after 6–8 weeks of fasting, the expression of AMPK increases by 50% in response to a GTT given to elephant seal pups, yet no change was observed in those fasting for only 2–3 weeks (34). Increased glucose uptake by a variety of tissues may be an important feature contributing to reduced circulating glucose concentration and decreased risk of metabolic syndrome. Increased glucose uptake leading to reduced circulating glucose concentrations and subsequent alleviation of the diabetic-like state has even recently been hypothesized to explain why gastric bypass surgery reverses diabetes in subjects before subsequent weight loss occurs (42).

### Cortisol

Elevations in cortisol have been implicated as a contributing factor to the development of the metabolic syndrome. Glucocorticoids, in particular cortisol, are known to enhance EGP and contribute to hepatic and extrahepatic insulin resistance (43), thus promoting hyperglycemia. Under acute elevations, cortisol is also known to facilitate lipolysis and affect the regulation of NEFA metabolism (44); however, under chronically elevated conditions, its role in regulating NEFA remains uncertain, although it may have different modes of action depending upon the specific adipose depot targeted (45).

Cortisol increases with time fasting in lactating females and weanlings (Table 1) and has been correlated with changes in peripheral insulin resistance within weanlings (34). Similar fasting-dependent variations in cortisol concentrations are not observed in adult males, although levels across the fast are similar to the highest levels observed in adult females and weanlings. Furthermore, cortisol is negatively correlated with body mass late in the breeding fast suggesting a potential relationship between endogenous nutrient reserves (i.e., adiposity) and cortisol production in competitively breeding males (33). In all cases, fasting levels exceed what might be considered “stressed” in humans. Patterns of circulating cortisol correlate well with the development of insulin resistance in elephant seals but elevations in cortisol are not correlated with an increase in EGP or circulating glucose levels.

### Non-esterified fatty acids

The role of NEFA in the development of insulin resistance has received much attention. Fasts as short as 24-h, which substantially increase circulating NEFA levels, have been shown to reduce both the acute glucose-stimulated insulin response and insulin sensitivity in human subjects (46). Both hepatic and peripheral insulin resistance are facilitated by elevations in NEFA and a recent review of the important role of adipose tissue in regulating metabolic pathways during fasting has recently been made (47). As in all mammals studied, NEFA increases with time fasting in the elephant seal (Table 1) and, like cortisol, NEFA has been related to changes in peripheral insulin resistance within this species (34). Measurements of lipolysis in lactating females suggests that the increase in NEFA with time fasting is not related to changes in lipolysis (14), leaving reduced uptake and decreased reesterification as mechanisms underlying the observed hyperlipidemia. In weanlings, no direct measure of lipolysis has been made, but an increase in adipose triglyceride lipase (ATGL) is suggestive of increased lipolysis with time fasting. However, reduced peripheral uptake of NEFA due to the depressed expression of fatty acid translocase (CD36), fatty acid transport protein (FATP1), or modulation of reesterification via PEPCK are also associated with increasing NEFA levels (36).

The role of obesity and fatty acids in the development of insulin resistance has been recommended for re-evaluation based on accumulating evidence that elevated NEFA is not necessarily related to insulin resistance and that insulin resistance can exist in obesity without elevations in NEFA (48). The distinctions of this debate will not be discussed here, but it is important to note several characteristics of elephant seal lipid stores and mobilization that might have relevance to the issue. First, lipid stores in the elephant seal are contained almost exclusively in the blubber and there is negligible visceral fat. The fat depot can approach 50% of the seal’s mass. Though obese by human standards, this level of adiposity is completely normal for this species; in fact, fatter seals are generally considered to have a better overall body condition. Second, the total lipid profile is markedly different within fasting adult males, which may or may not be reflected in the other age categories but which is certainly closer within the seals than in comparison to humans. Specifically, levels of total cholesterol and low density lipoprotein are elevated relative to humans but do decline with time fasting, from 10.2 to 6.9 mM and 4.2 to 2.0 mM, respectively (49). High-density lipoprotein is also high but is maintained across the breeding fast in males ranging from 4.6 to 5.7 mmol/L, well in excess of the threshold below which NCEP sets as one of the defining criteria of the metabolic syndrome (HDL ≤1.03 mM for males, ≤1.29 mM for females). Third, the fat depot is derived exclusively from the consumption of marine organisms and although the characterization of blubber stores has not been completed in adult elephant seals, the blubber layer of weanlings is dominated by the presence of short-chained mono-unsaturated fatty acids (SC-MUFAs) (50). The fatty acid composition of the blubber of the closely related southern elephant seal (*Mirounga leonina*) consists mostly of polyunsaturated fatty acids (PUFA) and both long and short-chained MUFA (51) and it is expected that the northern elephant seal would have similar overall distributions. Thus, it is likely that the profile of mobilized NEFA during fasting is substantially different than that of many humans experiencing or developing the metabolic syndrome, although the variability may be geographically dependent (i.e., diets of westernized cultures vs. Pacific island populations). This is important as certain NEFA constituents, particularly MUFA, have putative protective properties from cardiovascular disease and the prevention of the metabolic syndrome (52, 53). How the mobilization of NEFA and the overall lipid profile therefore relate to the adaptive nature of insulin resistance in the elephant seal remains unknown, but might provide useful insight into the pathology of insulin resistance in humans.

### Glucagon

It has been suggested that elephant seals do not regulate glucose via the typical insulin and glucagon push-pull model (38). However, significant relationships between proportional glucose cycle activity and the ratio of plasma insulin to glucagon (I:G) have been observed (23), and there is a consistent reduction in I:G between early and late fasting periods across all studies to date (Table 1). Specific action of either hormone is difficult to determine as any causative relationship to glucose kinetics is clouded by the fact that, although changes in I:G with time fasting are consistent, the changes in glucagon levels with time fasting are variable across studies and life history state of the seal.

### Adipocytokines

Several adipocytokines have been implicated in the development of insulin resistance in human populations. Leptin levels have been correlated with the metabolic syndrome and may vary as a function of ethnicity and gender (54–56). Conversely, low levels of adiponectin have been correlated with multiple physiological features of the metabolic syndrome as well as type 2 diabetes (57, 58). A recent investigation of adult men suggests that the ratio of adiponectin to leptin may be an important indicator of several factors related to the metabolic syndrome (59). Only a couple of studies have investigated variations in adipocytokines in elephant seals, but the results of these studies are enlightening.

Leptin levels in elephant seal weanlings do not change with time fasting or vary with changes in adiposity (34). In adult males, there is a small decrease in leptin with time fasting but no relationship to fat mass (33). Thus, there does not seem to be a relationship between variations in leptin and insulin resistance. Adiponectin significantly decreases and tumor necrosis factor-α (TNF-α) significantly increases across the fast in weanlings (34, 35, 60). The patterns of decreasing adiponectin, decreasing adiponectin/leptin ratio, and increasing TNF-α are consistent with increasing insulin resistance observed in this species. However, the relationship of either leptin or adiponectin to other cardiovascular risk factors typical of humans (e.g., total cholesterol) has not yet been determined in elephant seals, so it remains to be seen whether such relationships exist and how the expression of these compounds relate to the fasting capabilities of the elephant seal. The study of adipocytokines is relatively new in the elephant seal model, but future efforts will need to explore relationships of other adipocytokines to the physiological traits that characterize elephant seal fasting, particularly those adipocytokines that are thought to be offensive in humans when over-expressed by adipose tissue (e.g., PAI-1or visfatin).

## The Adaptive Value of High EGP and Insulin Resistance

Although fasting elephant seals demonstrate some characteristics that parallel those of the metabolic syndrome, particularly insulin resistance and a fasting hyperglycemia, it is necessary to return to the point that these characteristics are adaptive in this fasting-adapted species. Beyond the contribution of insulin resistance to diminishing glucose uptake and oxidation, which conserves valuable protein stores while fasting and promotes the use of copious lipid stores for meeting metabolic demands, other aspects of elephant seal physiology must be taken into consideration as they have bearing on the interpretation of their modifications to glucose metabolism. Of particular interest is the potential for elevations in EGP to reflect the influence of periodic hypoxic events associated with apneustic breathing on land and breath-hold diving, as well as the prevention of ketoacidosis while fasting. These factors have been previously discussed (24) but are presented again here for the sake of providing a more complete picture of the adaptive potential of fasting glucose metabolism in the elephant seal.

Some aspects of elephant seal fasting metabolism may reflect adaptations to mitigating hypoxia and which potentially explain deviations from characteristics of the metabolic syndrome. Hypoxia is known to affect lipid and carbohydrate metabolic pathways and elephant seals are repeatedly exposed to hypoxic conditions and tissue ischemia because of their continuous breath-hold diving while at sea and their apneustic breathing patterns while on land (61–64). For example, hypoxia inducible transcription factor (HIF) is responsible for mediating the effects of hypoxic-ischemic stress. In low oxygen conditions it ceases to be a target for enzymatic hydroxylation by intracellular oxygen sensors, called prolyl hydroxylases (PHD), enabling it to transactivate the genes responsible for adapting cellular pathways to hypoxic conditions. Furthermore, selective disruption of certain hydroxylases, specifically PHD-1, have been shown to further up-regulate HIF expression. These conditions subsequently increase the use of anaerobic pathways and affect various aspects of carbohydrate metabolism, such as reducing pyruvate conversion to acetyl CoA, suppressing its entrance into the TCA cycle, and increasing levels of glycolytic enzymes and lactate dehydrogenase (65, 66). Although many aspects of elephant seal glucose metabolism are consistent with the upregulation of HIF, it remains to be determined as to how or if adaptations to repeated hypoxic conditions have coevolved with the need to conserve endogenous nutrients during fasting such that the typical mammalian response to prolonged food deprivation has been modified (67, 68).

Another possible explanation for the fasting hyperglycemia and high rates of EGP is that they are consequences of an up-regulated TCA cycle for purposes of preventing ketoacidosis (15). Although indices of incomplete NEFA β-oxidation (C6–C14 carnitines) increased with time fasting in weanlings (36), there is only a modest accumulation of ketones (i.e., no ketoacidosis), and this despite elephant seals meeting ∼90% or greater of their metabolic demand through fat catabolism (13, 15, 17). During long duration fasts undertaken by non-fasting-adapted species significant ketogenesis can occur, and in diabetes mellitus ketoacidosis can occur as a pathological response to continued elevated rates of lipid mobilization. The speculated cause of this phenomenon is that the rate of fatty acid β-oxidation provides acetyl CoA at rates that exceed the processing capacity of the TCA cycle. That an up-regulated TCA cycle accommodates a high rate of fatty acid β-oxidation in elephant seals is supported by NMR spectroscopy that shows pyruvate cycling and the flux of carbon through PEPCK are elevated relative to other mammalian species (21). Thus, whereas hepatic and peripheral insulin resistance facilitates lipolysis and glucose production, the latter may be further enhanced via modifications to anaplerotic pathways supporting the mitigation of ketosis.

## Evolutionary Convergence Among Marine Mammals?

**Figure 2:**
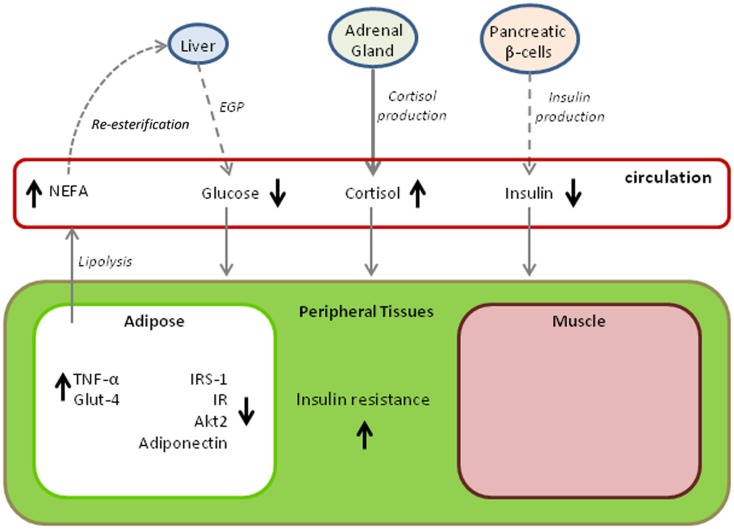
**Variations in metabolic pathways with time fasting that contribute to insulin resistance in the northern elephant seals**. The model draws upon findings from multiple age classes and likely demonstrates age and gender-specific variations. Dashed lines indicate reductions in metabolite or hormone production, bold lines indicate increased production, and normally weighted lines indicate no change. Arrows next to a metabolite or hormone indicate either increased levels (upward arrow) or decreased levels (downward arrow). (EGP, endogenous glucose production; NEFA, non-esterified fatty acids; TNF-α, tumor necrosis factor alpha; IRS-1, phosphorylated form of insulin receptor substrate; IR, phosphorylated form of insulin receptor; Akt2, expression of the Akt2 gene; Glut-4, expression of the plasma membrane glucose transporter 4.)

Figure 2 provides a summary of changes in metabolic pathways that occur with the progression of fasting in elephant seals. The diagram is somewhat hypothetical in that it combines findings from different age classes of seal, but it also demonstrates adaptive variations that may be shared with other marine mammals. A common factor among all cetacean and pinniped species is that they have evolved a secondary aquatic existence over tens of millions of years while capitalizing on a diet that is high in lipid and protein content but essentially devoid of carbohydrate. The result of this dietary pressure may very well have produced a convergence of metabolic adaptations that parallel those of pathological states in terrestrial mammals, particularly in humans where an overabundance of energy rich foods taxes a system originally designed for dealing with food shortages. The metabolic characteristics of prolonged fasting in the elephant seal described here are not unique to this species. Evidence obtained from other marine mammals, including odontocete cetaceans (e.g., toothed whales such as dolphins) and both otariid and phocid seals (i.e., sea lions and fur seals, and true seals, respectively), suggests that the same traits are expressed to some degree within many species of these divergent, phylogenetic lineages. For example, high fasting blood glucose is observed in a number of pinnipeds with widely varying fasting durations (69–72) and is even observed in dolphins fasting for periods of several days (73). Other physiological parallels to the metabolic syndrome and diabetes mellitus have also been noted (74); however, the degree to which there are parallels varies according to the physiological ecology of the species. For example, pups of both the subantarctic fur seal (*Arctocephalus tropicalis*) and the northern elephant seal undergo extreme fasts of up to 3 months, but the hormonal regulation of substrate utilization during the fasts is markedly different, likely because of the more restricted nutrient reserves in the smaller subantarctic fur seal (75).

## Summary

The physiological response to fasting shared by mammals produces a state of insulin resistance that helps to conserve endogenous protein stores from net loss via gluconeogenesis and glucose oxidation and facilitates lipolysis in order to increase the reliance upon fat oxidation to meet the organism’s metabolic demands. In animals adapted to long-term fasting and which predictably incorporate fasts as part of their life history, the physiological modifications to fasting can be taken to the extreme. Conversely, in the modern human era, chronic energy surplus combined with reduced physical activity has resulted in a set of conditions that contribute to insulin resistance and increased morbidity, mainly excess fat deposition, lipid overflow into non-adipose tissues, and subsequent inflammatory responses. In the former condition the role of insulin resistance is adaptive, whereas in the latter it is pathological. Investigations of insulin resistance in fasting-adapted marine mammals, such as the northern elephant seal, might provide insight into the pathophysiology of insulin resistance in humans and how it might be better treated through pharmacological approaches. Given that the marine mammals have evolved over millions of years to live off of high energy diets rich in fat and protein and nearly devoid of carbohydrate, it seems that a number of lessons regarding the issue of the metabolic syndrome in humans might be learned.

## Authors Contribution

This review article was co-written by Dorian S. Houser, Cory D. Champagne, and Daniel E. Crocker.

## Conflict of Interest Statement

The authors declare that the research was conducted in the absence of any commercial or financial relationships that could be construed as a potential conflict of interest.
